# Dr. Walker, on the Use of the Spleen

**Published:** 1807-02

**Authors:** John Walker

**Affiliations:** Salisbury Court


### *To the Editors of the Medical and Physical Journal.*

GENTLEMEN,

WHILE your Journal continues to exhibit papers of physiologists so ingenious, and so eminent, as Dr. Rush and Mr. Saumarez, *cum multis aliis,* the most fastidious of your readers will perhaps bear, with a patient smile, the never ending war of words on horses' heels, even by those who know nothing about them, as well as on the simple subject of Cow-pox, as produced upon the human subject, and now, happily, pretty well understood, in every quarter of the globe; though, the very first and the most most prolix writers on it, in this country, are some of the most inconsistent characters to be met with, in their conduct as to its propagation.

Having read, in your late numbers, with considerable interest, both Rush and Saumarez, on the chylopoetic viscera, &c. I could not help being a little struck with their ardour, in support of their respective systems. Do they not both, sometimes, enlist into their service certain arguments; and do they not both sometimes make statements of facts, which yield not any strength to their different causes? (Medical and Physical Journal, Vol. xvi. p. 194.)

In the artery of the spleen, being rather stronger than the aorta, there may be an admirable provision for preventing a continual and needless expansion of this viscus. It only yields to enlargement, when the excessive force of the systole produces a sort of general reaction, from the whole arterial system, seasonably opening this stronger artery, as a waste gate. But how do the veins, possessing a peculiar and specific strength, assist in the work? In asking the question, it occurs to me that the Doctor's idea must have been, that the temporary yielding of the artery to the increased influx of blood, and the permanent resistance of the inelastic veins, to much more than the ordinary egress of it, are, unitedly, the cause of the seasonable enlargement of this spongy and distensible viscus. In page 195, he exhibits some facts which I think strikingly illustrative of the justness of his opinion. The spleen, by its organization, seems only to be the slave destined to endure the effects of the irregularities of its superiors, and therefore it possesses but little sensibility.

If the blood of the spleen be less coagulable than that obtained by bleeding, or from wounds, how can this peculiarity have been produced, by its having been thrown with force into that organ, as well as by its being feebly propelled back again into circulation? The former would be a cause of greater coagulability, by bringing it most speedily, in its ameliorated state, from the heart and lungs; the latter only, a cause of less coagulability, by detaining it from the place of aerial renovation. The force of a blow on the stomach does, with the deprivation of life of the animal, produce incoagulability of the whole mass; but surely there is no sort of analogy between such lethal violence, and the increased force from a quickened circulation.

P. 196, Dr. Rush offers, in support of his opinion, the

 nature nature of the diseases which produce obstructions in the spleen; as, yellow fever, bilious tertians, dysentery, chronic fevers, cases of pulmonary consumption, and even mania; malignant passions also, whatever they may be, are considered, from the figurative expression splenetic, as affecting this waste gate of the system. Is not all the support of his opinion, which he derives from disordered spleen, under diseases so different, altogether hypothetical? Is not that which he derives from the diseases following the loss of the spleen; as enlarged liver, indigestion, head-ach, and increased secretion of saliva, urine, and semen, equally so? But, his accounting for the cases of sudden death, exhibiting the spleen unenlarged; and for the difference of the spleen in the human species, from that of brutes; (consisting chiefly in a texture, calculated to afford more promptly and freely, a receptacle for the blood, when exerted into tumultuous motions, through the medium of our greater portion of mind) seems felicitously in accordance with his theory. Even his referring to this viscus, the absorbing and suffocating of undue impressions, upon the nervous system and the mind, however fanciful, is perhaps correct, and certainly ingenious.

Waving, very much, the attempt to enter into the consideration of the different physiological and pathological doctrines of Dr. Rush and Mr. Saumarez, which I hope will be taken up by some of your well-informed readers of leisure, I yet think it very fair to adopt the more humble task of endeavouring to find out any obvious deficiencies in their arguments. It is peculiarly important, that the eminent should be guarded in their communications to the public. Their theories should always be well examined; or, otherwise, we might often be induced to receive mere crudities for ripened fruits.

P. 200. Venous blood, from which the hepatic bile is secreted, is less disposed to putrefaction than arterial blood, taken from any other part of the body. 'I am disposed to ascribe it, chiefly, to its containing a quantity of imperfect chyle, which we know passes more slowly into a putrid state than blood.'—Here, the Doctor seems not to have recollected that the 'imperfect chyle' first pervaded the arterial current; but perhaps he may intend, in the comparison, venous blood soon after a full meal, and arterial blood of later pulsations, when the chylous particles may have chiefly found their way to the liver, and already been subjected to a secretory process in that organ.

P. 203.

P. 203. And when the chylopoetic office of the liver 'is impaired or interrupted, the stomach, by performing its functions with double care, prevents the evils of an abstraction of nourishment from the body.'—Must not this conclusion appear altogether hypothetical? Does the stomach at any other time perform its functions so well, as when the liver is in perfect health?—"It has often been remarked, p. 205, that the digestive process, in the stomach, required a healthy and tranquil state of the whole system."

P. 204. "Had it been otherwise, (i. e. had not the liver received the blood from every part of the body, in order to subject that part of it, which had not been completely animalised, or divested of its chylous properties, to a secretory process, and afterwards to pour the product of this secretion, mixed with the liquor of the pancreas, into the duodenum, to be absorbed, or otherwise taken up by the lacteals, and conveyed with the chyle from the stomach into the blood-vessels) all that portion of chyle which was incapable of being made into blood, by passing through the lungs, would have been lost to the system."—Why should it have been lost? If the lungs be the place for the sanguification of chyle, would not the chylous particles, distributed through the whole mass of blood, be quite as certainly secured to the system, on their future arrival at the lungs, as on their arrivals at the liver, when they happened to reach it?

P. 289. Mr. Saumarez, with a view to avoid beginning the discussion of Dr. Rush's doctrines in the middle, as their author has done, says, it will be necessary to invert the order in which he has placed them; that is, begin at both ends, and finish in the middle. I make this observation on his random statement, at the commencement, to shew, that while I often have the pleasure of hearing him, in the discussion of medical subjects, and find him as unceremonious a disputant, as the most worthy cause could ever require; I also am sensible of that excellent disposition in himself, he is never offended at being told, *tout bonnement*, that he is certainly wrong, if his opponent sincerely think so.

Supposing Mr. Saumarez's own ideas on the evolution of the fœtus in utero to be clear and correct, what can he mean, p. 295, by the blood which is effused from thence, (the maternal portion of the placenta?) on the foetal portion, (and which resembles in some respect chyle,) is the

 power power by which it becomes assimilated, &c.? What impenetrable obscurity hangs over this statement! Is the effused blood, resembling chyle, the power by which itself becomes assimilated? To speak of the active energy of a fluid as well as of an organ, affecting other matter, page 290, is not, I think, the usual mode of expression in physiology; but, to talk of the blood being the power, by which it is presumed it (the blood!) becomes assimilated, &c. reminds one of the old Merry-Andrew expression, so diverting to school-boys, of a man's leaping down his own throat.

Had there been a free uninterrupted passage of the parental, arterial blood, through the placenta, then, notwithstanding 'voracity of appetite and comparative weakness of organs,' the 'auxiliary means' along the 'chylous canal,' with the 'digestion of the air,' and amelioration of the circulating mass, on the passage through the lungs, might have made all appear pretty simple. We might have seen in the 'pure and vital nature of the arterial blood' of the parent, some explanation of the evolution of the fœtus by analogy with what takes place in the continued repair of our own bodies. But, the incipient, tender embryo, the matured fœtus, even, is not to be subjected to the irregular actions of the parent heart. A stop gate is effectually opposed to them in the placenta. Thro' this barrier the vessels of which cannot be permeated after death by the finest injections, the necessary pabulum of the fœtus, whatever aura or fluid it may be, does by the sympathising, harmonising action in living parts, find its way, and is elaborated into all the varieties of form and substance, which make up our organization. Mr. Saumarez has not any knowledge whatever, of the different degrees of evolution, which take place in the different organs of the fœtal frame; he conceives not the shadow of an idea of how they are effected; nor yet how they escape becoming entirely moribund, while they are so far, and so effectually secluded from the direct product of his digestion of air in the lungs; which designation, however, in a word of the business of respiration, is perhaps more apposite and certainly more comprehensive, than all the conjectures of the chemists, with their different agents, sensible or latent, gaseous or concrete. P. 296, he says, the low degree of vitality, which venous blood contains, that renders bile out of which it is formed, (which is formed out of it) to possess less living, and consequently, more sensible properties, than either the gastric or pancreatic juices,'

juices,' 'that the farther hepatic bile proceeds from its source, either when subsisting in the gall bladder, or when united with fœculent matter in the intestinal canal, that (the more) it verges from a living to a dying state, that (the more) it loses its innoxious, and acquires its sensible and stimulating qualities,' &c.—Do its sensible qualities render it a less active chemical agent? P. 293 'we may infer, from the pure and *vital* nature of the arterial blood, of which the pancreatic juice is produced, and from the more direct termination of the duct itself, that it forms a more *active*, and more constant *precipitant*, than the fluid produced by the liver.'—But does Mr. Saumarez give to the bile, a duty less active, than to the secretion of the pancreas?—'We may conclude, I say, p. 293, that the pancreatic juice separates the less feculent parts of the chyle from the most pure; and, finally, that the bile separates the most feculent of the whole from the less offensive part, completing what the pancreatic juice had begun.'—Here, the bile becomes to the pancreatic fluid, what Dr. Rush, p. 204, makes the liver become to the stomach; 'a second bolting cloth to a mill; though for a directly contrary purpose; an accomplisher of the work,' which the first, and constantly present agent, had left incomplete. I think Mr. Saumarez will not be able to exhibit any chemical process out of the living body, analagous to his explanation of what passes in the chylous and excrementitious canal; but, granting him, in unorganized parts, in fluids produced by secretion, more or less possession of vitality, his doctrines then may, perhaps, become susceptible of a perfect comprehension. The pancreatic juice, very naturally, from its superior vitality, makes selection of the purest part of the chyle, throws it down, and, perhaps, itself along with it, on the surface of the chylous canal, hereby securing to itself, a passage through the lacteals, and a future life; while the poor bile being rather later in arriving at the scene of action, does, in effecting further separation of the nutritious parts from the feculent, become so involved with the latter, as to get completely swept off, with them, in the retreat.

P. 300. 'Whenever the stomach is full, the pressure which it produces on the spleen, not only prevents the passage of blood from the splenic artery, through that organ, but does actually cause an increased accumulation in the vessels, which the splenic trunk gives out to the stomach and pancreas. If this be the case, the liver must also receive a larger quantity of blood from the vena portæ, for the secretion of the bile, as it is principally made up of the veins, which return the blood from those organs.' Not by the pressure of the stomach, if it prevents the same quantity of blood from entering the spleen, which it caused accumulation of, in the vessels of the stomach and pancreas.

There is one part of the paper, in which Mr. Saumarez seems to rise very high in the contemplation of his subject. It is where he discovers a warfare of parts in the doctrines of other physiologists. P. 297, 'The chylous canal is especially constructed by the number and transversion of valvulæ conniventes, to *retard* the motion of the chyme, along the first part of its course; it is therefore most unreasonable to suppose, that the real and direct intention of the hepatic system, is to hasten its expulsion; if this were the case, no harmony between these parts could subsist: the action of the chymous canal would always tend to prevent the motion which the bile was always destined to promote, &c.' P. 298, 'Men who reflect on the physiology of the animated system, and can reconcile this warfare of parts, that are dependent on each other, possess very inadequate and erroneous phantasies of the beautiful harmony and subordination, that subsists between the parts of which the whole is composed.' Here Mr. Saumarez seems to have forgotten that the whole order and harmony of creation, is supported by what he calls a warfare of parts. The planets are constantly subjected to certain forces. The centrifugal, constantly tends to throw them off, in a right line, into the immensity of space; the centripetal, to plunge them directly into the sun; and by this warfare of parts, their orbits are produced. By the exhalants moisture is supplied throughout every part of the system; and from every part of that system the absorbents continually take up fluids. By the adductor and flexor muscles, certain parts are liable to be contracted into deformity; the abductors and extensor are their *antagonists*, and by this never ceasing warfare of parts, order, beauty, and harmony, are preserved.

JOHN WALKER

*Salisbury Court, 27, xij,* 1806.

